# Diagnostic Performance of the Fujifilm SILVAMP TB-LAM in Children with Presumptive Tuberculosis

**DOI:** 10.3390/jcm10091914

**Published:** 2021-04-28

**Authors:** Patricia Comella-del-Barrio, Bárbara Molina-Moya, Jacqueline Gautier, Raquel Villar-Hernández, Mariette Jean Coute Doresca, Beatriz Sallés-Mingels, Lydia Canales-Aliaga, Margareth Narcisse, Tomás M. Pérez-Porcuna, Jacob Creswell, Luis E. Cuevas, José Domínguez

**Affiliations:** 1 Institut d’Investigació Germans Trias i Pujol, CIBER Enfermedades Respiratorias, Universitat Autònoma de Barcelona, 08916 Barcelona, Spain; patricia.comella@e-campus.uab.cat (P.C.-d.-B.); bmolina@igtp.cat (B.M.-M.); raquel.villarhernandez@gmail.com (R.V.-H.); 2 Department of Pediatrics, Division of Tuberculosis, Hôpital Saint-Damien, Nos Petits-Frères et Sœurs, Port-au-Prince 6112, Haiti; jacqueline.gautier@nph.org (J.G.); mamaille71@yahoo.fr (M.J.C.D.); margareth.narcisse@nph.org (M.N.); 3 Department of Radiology and Imaging Diagnose, Centre d’Atenció Primària (CAP) Manso, 08015 Barcelona, Spain; reyes.salles@gmail.com; 4 Servei de Pediatria, Atenció Primària, Unitat de Investigació Fundació Mútua Terrassa, Hospital Universitari Mútua Terrassa, 08221 Barcelona, Spain; lcanales@mutuaterrassa.es (L.C.-A.); tomas.perez.porcuna@gmail.com (T.M.P.-P.); 5 Stop TB Partnership, TB Reach, 1218 Geneva, Switzerland; jacobc@stoptb.org; 6 Department of Clinical Sciences, Liverpool School of Tropical Medicine, Liverpool L3 5QA, UK; Luis.Cuevas@lstmed.ac.uk

**Keywords:** tuberculosis, diagnosis, lipoarabinomannan, LAM, children, urine, point-of-care

## Abstract

Current diagnostics for tuberculosis (TB) only manage to confirm a small proportion of children with TB and require respiratory samples, which are difficult to obtain. There is a need for non-invasive biomarker-based tests as an alternative to sputum testing. Fujifilm SILVAMP TB lipoarabinomannan (FujiLAM), a lateral-flow test to detect lipoarabinomannan in urine, is a novel non-sputum-based point-of-care diagnostic reported to have increased sensitivity for the diagnosis of TB among human immunodeficiency virus (HIV)-infected adults. We evaluate the performance of FujiLAM in children with presumptive TB. Fifty-nine children attending a paediatric hospital in Haiti with compatible signs and symptoms of TB were examined using Xpert MTB/RIF, smear microscopy and X-rays, and classified according to the certainty of diagnosis into bacteriologically confirmed TB (*n* = 5), unconfirmed TB (bacteriologically negative, *n* = 50) and unlikely TB (*n* = 4). Healthy children (*n* = 20) were enrolled as controls. FujiLAM sensitivity and specificity were 60% and 95% among children with confirmed TB. FujiLAM’s high specificity and its characteristics as a point-of-care indicate the test has a good potential for the diagnosis of TB in children.

## 1. Introduction

Tuberculosis (TB) is a major cause of morbidity and mortality in children [1]. However, the paucibacillary nature of TB in children and the difficulty of obtaining respiratory samples create significant barriers for diagnosis [2]. Most children with TB, especially in countries with high TB burden [3], are not bacteriologically confirmed, or are missed [3]. The lack of reliable diagnostic methods has highlighted the need for non-sputum-based tests for childhood TB [4,5].

In recent years, attempts have been made to develop diagnostic tests based on the detection of lipoarabinomannan (LAM) antigen, a lipoglycan and virulence factor in the bacteria genus *Mycobacterium* present in the outer cell wall, which is released from metabolically active or degenerating bacterial cells. As LAM is heat stable, filtered by the kidney and detectable in the urine of people with overt TB, test prototypes have attempted to identify LAM in urine since the 1930s [6,7]. Until recently, urine-based LAM prototypes have had low sensitivity and performed better in individuals co-infected with human immunodeficiency virus (HIV) and advanced immunosuppression [8]. Although the biological mechanisms for the better performance in HIV-positive individuals are not fully understood, the higher concentration of LAM in the urine could be due to a greater difficulty in containing *Mycobacterium tuberculosis* with subsequent hematogenous spread to the kidneys or to a higher body burden of *M. tuberculosis* [9,10,11,12]. Compared to the Alere Determine TB-LAM assay, with a reported sensitivity of about 40% for detecting TB in HIV-infected patients [13], a new assay is reported to have higher sensitivity to detect LAM in both HIV-infected and uninfected patients [14,15]. The assay, the Fujifilm SILVAMP TB LAM (FujiLAM, Fujifilm, Tokyo, Japan), combines high-affinity monoclonal antibodies against *M. tuberculosis*-specific LAM epitopes and a silver amplification step to increase the visibility of the test and control lines [15]. However, data on its performance in children is limited, and we report here its performance in a case series of children with signs and symptoms of presumptive TB. TB in children has more disseminated clinical presentations than adults because of their poor containment of TB. In addition, children have difficulty in expectorating sputum, and assays that use non-sputum samples could be useful, particularly in young children [2,11,16,17,18].

## 2. Materials and Methods

This was a retrospective case series of the bio-banked urine samples of 59 children with signs and symptoms compatible with TB, and of 20 healthy control children. Children were enrolled prospectively between August 2015 and December 2016 [19]. The children with presumptive TB were between 0 and 14 years old, and were enrolled when attending the Saint Damien paediatric reference hospital in Port-au-Prince, Haiti. Children were initially identified by the diagnostic clinics and enrolled opportunistically. This is a case series, as we did not estimate the number of children required and instead enrolled as many children being investigated for TB as possible over a period of sixteen months. The healthy children, enrolled as controls, were attending a primary school in the same neighbourhood of the hospital and were between 5 and 8 years old. The objective of including a control group was to explore whether any of the children without symptoms of TB had a positive FujiLAM test to better characterize the specificity of the test.

After obtaining informed parental consent, we collected clinical and demographic data, vaccination history (including Bacillus Calmette-Guérin (BCG)) and information about current and previous medications. Children were excluded if they had known immunodeficiencies, were receiving immunosuppressive treatment, or if they had received anti-TB treatment for two or more weeks before enrolment. Tuberculin skin tests (TSTs) were performed by a trained technician using 0.1 mL of Tubersol (bioequivalent to 5 Tuberculin Units; Sanofi Pasteur, Canada) and were considered TST positive if they had ≥10 mm induration in the presence of a BCG scar or ≥5 mm in non-BCG-vaccinated children with no known contact with adults with TB [20]. Three millilitres of blood was obtained for the QuantiFERON-TB Gold In-Tube test (QFT-GIT, Qiagen, Germany [21]), following the manufacturer’s instructions.

Children with presumptive TB underwent anterior–posterior chest X-rays, which were read by two radiologists blinded to the child’s condition, and a third reader resolved reading disagreements. Three consecutive sputum samples obtained by induced or nasopharyngeal/nasogastric aspiration were examined by fluorescence smear microscopy stained with auramine, and children with positive smear microscopy or abnormal X-rays underwent Xpert MTB/RIF (Cepheid, Sunnydale, CA, USA) following local diagnostic algorithms. No culture facilities were available. Children with lymph node adenopathy underwent biopsies for histological examination.

Children with presumptive TB were classified using the clinical case definitions into confirmed, unconfirmed, and unlikely TB [22]. Children were classified as having confirmed TB if bacteriologically confirmed by Xpert MTB/RIF; as unconfirmed TB if there was no bacteriological confirmation but a positive TST or QFT-GIT and at least one of the clinical criteria of the Clinical Case Definitions (i.e., X-rays consistent with TB, signs and symptoms of TB, close TB exposure, or positive response to TB treatment), or at least two clinical criteria if the TST and QFT-GIT results were negative; and unlikely TB if the child had only evidence of *M. tuberculosis* infection or presented only one clinical criterion compatible with TB. Enrolled school children had a negative TST and QFT-GIT, and no signs or symptoms of TB. Those with positive TST or symptoms compatible with TB were referred to the hospital for TB screening and were not included as controls.

Participants were asked to provide a midstream urine sample on-site, collected in sterile plastic containers, or in urine bags for children <1 year, and stored at room temperature if they were processed at the time of collection, or stored in a refrigerator if processed in batches at the end of the day. Urine samples were aliquoted (2 mL) into cryovials and kept at −20 °C until processing in Badalona, Spain (IGTP). To test with FujiLAM, urine samples were thawed at room temperature, mixed with a vortex, and processed following the manufacturer’s instructions steps: first, the reagent tube was filled with urine up to the indicator line, mixed and left to incubate for 40 min at room temperature. The tube was then mixed, two drops of urine were added to the test device, and the first button was pressed. After 3–10 min of incubation, the second button was pressed to release the silver ions around the gold-conjugated antibody. The results were then read after one minute by two investigators blinded to the participants’ TB status, following the manufacturer’s instructions. If readers disagreed on the presence of test lines, the test was repeated once.

Categorical variables were described by frequencies and percentages, and quantitative variables described using means and 95% confidence intervals (CIs) if normally distributed, and medians and interquartile ranges (IQRs) if not normally distributed. We used Fisher’s exact test, chi-square and Kruskal–Wallis tests to compare between groups, and *p*-values < 0.05 were considered statistically significant. The sensitivity, specificity, and predictive values of FujiLAM were estimated using Xpert MTB/RIF as the microbiological reference standard, and against a composite of the combination of clinical diagnosis and Xpert MTB/RIF. Diagnostic accuracy was calculated for microbiologically confirmed cases vs. cases with unconfirmed TB, unlikely TB or controls, and for the composite reference standard vs. unlikely TB and controls. Statistical analysis was performed using SPSS (SPSS version 26.0, SPSS Inc, Chicago, IL, USA). The World Health Organization (WHO) Anthro 3.2.2 (<5 years) and Anthro Plus 1.0.4 (≥5 years) were used to estimate *z*-scores [23,24]. Undernutrition was classified as a weight-for-age *z*-score < −2 (or as body mass index-for-age *z*-score < −2 for children above 10 years) and stunting as a height-for-age *z*-score < −2.

Informed consent was obtained from the parents or legal guardians. The study was approved by the Ethics Committee of the University of Barcelona and the Haiti National Ethics Committee (reference number IRB00003099).

## 3. Results

Eighty-two children were enrolled, of which three were excluded due to insufficient urine volumes. Of the 59 with presumptive TB, 5 were bacteriologically confirmed, 50 had unconfirmed but clinically diagnosed TB, and 4 were deemed not to have TB. The additional 20 were healthy controls. Demographic characteristics of the study groups are shown in Table 1. Fifty-one (64.6%) children were male, with a median age of 76 (IQR 58–121) months. Their median body mass index was 14.80 (SD ± 2.4); eighteen (30.5%) reported recent and severe weight loss and twelve (20.7%) reported stunted growth. Fifty-six (75.7%) had received the BCG vaccine and had a BCG scar. FujiLAM results are shown in Table 2. Eight (10.1%) samples were positive and seventy-one (89.9%) negative (Table 2). Three of the positive samples belonged to children with bacteriologically confirmed TB, three to children with unconfirmed TB, one to a child with unlikely TB and one to a control. Among the six FujiLAM-positive children with bacteriologically confirmed and unconfirmed TB, four had intrathoracic and one both intrathoracic and TB spondylitis. If Xpert MTB/RIF was considered the reference standard, the sensitivity of FujiLAM was 60% (95% CI 17–93) and the specificity, considering the control group, 95% (95% CI 73–100). If confirmed and unconfirmed TB were compared against unlikely TB and controls, the sensitivity was 11% (95%CI 5–23) and the specificity 92% (95% CI 72–99). Five (71.4%) of the seven FujiLAM-positive children with presumptive TB were underweight and four (66.7%) were stunted (*p* = 0.023 and *p* = 0.014, respectively). The one case of disagreement of FujiLAM assays between the two readers was resolved after repeating the test.

## 4. Discussion

We evaluated the FujiLAM test in a case series of children attending the hospital with signs and symptoms of presumptive TB. Among children with bacteriologically confirmed TB, the test had a sensitivity of 60%, with a high specificity of 95%. However, the case series is small, which resulted in wide and overlapping 95% CI, and therefore larger studies are needed to confirm our findings. The current LAM assay (AlereLAM) recommended by the WHO in adults [8] and children [25,26] is only recommended for severely immunosuppressed adults with HIV and CD4 counts <100 cells/µL [8]. Therefore, a test that performs well in HIV-uninfected patients would be a significant improvement. Unlike its predecessor, FujiLAM combines a pair of high-affinity monoclonal antibodies targeting largely *M. tuberculosis*-specific LAM epitopes and a silver amplification step to improve the visibility of the test and control lines, allowing the detection of much lower LAM concentrations in urine [6,15]. Furthermore, the FujiLAM test identified three more children who had a clinical diagnosis of TB but could not be bacteriologically confirmed, which would have lent more support to the decision to treat.

Children have a high risk of developing disseminated forms of TB, and the hematogenous and lymphatic spread of *M. tuberculosis* is likely to result in high amounts of LAM in urine [2,9]. However, few studies have reported the performance of FujiLAM in children [27,28]. A multicentre study in four African countries reported a relatively high sensitivity of 67.5% in HIV-negative children [28], but a cohort study in South Africa reported a lower sensitivity of 38.8% [27]. The authors concluded that the differences in their results may have been due to the severity of the TB episodes, and that some of the studies included a high proportion of malnourished children [28]. It has been reported that LAM detection in urine is associated to the overall amount of *M. tuberculosis* in the body, as well as the severity of the disease [10,29]. In our study, sensitivity was higher among bacterially confirmed cases, who are likely to have more advanced disease stages, while sensitivity was lower when children with a clinical diagnosis were included. It is unlikely that all children with a clinical diagnosis of TB had TB (and thus some were misclassified), and it is thus difficult to classify a negative FujiLAM test as false negative. Furthermore, the number of positive cases was proportionally higher among underweight and stunted children [30,31,32]. Malnutrition has an impact in the several faces of the immune response against infections, including TB; this limits the contention capacity of the bacilli, increasing the amount of LAM in urine samples and, therefore, the sensitivity of the LAM tests [28].

FujiLAM specificity was high, which is consistent with previous findings in adults and children using microbiological (95%) and composite reference standards (91.7%) [10,27,28,33,34]. Although the test did not reach the 98% specificity recommended by the WHO, our sample size is not sufficiently powered to make this distinction and the 95% CI overlap with this value [35].

The FujiLAM’s main advantage is its urine-based format, making an easy to do and non-invasive test without the need for additional instrumentation [10], generating opportunities to diagnose TB in patients who do not excrete bacilli, such as people with extrapulmonary or military TB, HIV and children [4]. Especially among children, the test may be able to add value to clinical decision-making as a rule in test for TB, in our study effectively doubling the number of children treated who had confirmation of TB disease. To our knowledge, there are no studies evaluating the economic impact of LAM testing within the diagnostic algorithms for paediatric TB [36]. The inclusion of a test with the performance of the FujiLAM assay could simplify these algorithms and potentially diagnose a greater number of children with TB and increase the number of children initiating specific therapy [37]. Our study has significant limitations, including its small sample size—especially the small number of children with bacteriologically confirmed TB, which resulted in underpowered estimates. In addition, we did not culture the samples, and Xpert MTB/RIF and clinical diagnosis were used as reference standards. Xpert MTB/RIF is known to have lower sensitivity than culture [22], and thus it is likely it underestimated the number of children with bacteriologically confirmed TB. Other limitations concern retrospective testing of stored samples; however, studies using the FujiLAM test have shown good agreement between fresh and frozen urine samples [31,38].

In conclusion, FujiLAM had moderate sensitivity and high specificity in children compared to Xpert MTB/RIF, can process samples that are easier to collect than sputum, and can add a level of certainty to clinical diagnosis. These performance characteristics and the test’s operational characteristics would facilitate a rapid diagnosis in non-sputum samples in children—especially in low- and middle-income countries, where facilities available are limited. More extensive evaluations in children are warranted.

## Figures and Tables

**Table 1:** Demographic and clinical characteristics of participants.

	Overall (*n* = 79)	Confirmed TB (*n* = 5)	Unconfirmed TB (*n* = 50)	Unlikely TB (*n* = 4)	Controls (*n* = 20)	*p*-Value
Female	28 (35%)	1 (20%)	21 (42%)	1 (25%)	5 (25%)	0.530
Male	51 (65%)	4 (80%)	29 (58%)	3 (75%)	15 (75%)	
Median age (IQR) months	76 (58–121)	95 (51–128)	76 (51–122)	152 (98–165)	70 (58–94)	0.109
<5 yrs.	24 (30%)	1 (20%)	16 (32%)	0 (0%)	7 (35%)	0.654
≥5 yrs.	55 (70%)	4 (80%)	34 (68%)	4 (100%)	13 (65%)	
BCG scar (*n* = 74)	56 (76%)	3 (60%)	35 (78%)	2 (50%)	16 (80%)	0.390
TST or QFT-GIT positive	55 (93%)	5 (100%)	46 (92%)	4 (100%)	0 (0%)	1.000
TST positive	52 (88%)	4 (80%)	44 (88%)	4 (100%)	0 (0%)	0.707
QFT-GIT positive (*n* = 53)	37 (70%)	5 (100%)	28 (64%)	4 (100%)	0 (0%)	0.101
TB contact	50 (85%)	1 (20%)	45 (90%)	4 (100%)	0 (0%)	0.002
Cough	48 (81%)	5 (100%)	39 (78%)	4 (100%)	0 (0%)	0.491
Fever	40 (68%)	4 (80%)	34 (68%)	2 (50%)	0 (0%)	0.718
Lethargy	4 (7%)	3 (60%)	1 (2%)	0 (0%)	0 (0%)	0.002
Weight loss (*n* = 56)	19 (34%)	3 (60%)	15 (32%)	1 (25%)	0 (0%)	0.517
Adenopathy	22 (37%)	4 (80%)	18 (36%)	0 (0%)	0 (0%)	0.046
Underweight (*n* = 74)	18 (31%)	3 (60%)	15 (30%)	0 (0%)	0 (0%)	0.201
Stunted (*n* = 58) ^a^	12 (21%)	2 (40%)	9 (18.4%)	1 (25%)		0.418
X-ray consistent with TB	28 (46%)	5 (100%)	23 (46%)	0 (0%)	NA	0.161
Positive smear-microscopy (*n* = 52)	10 (19%)	3 (60%)	7 (16%)	0 (0%)	0 (0%)	0.062
Treatment completed	50 (85%)	5 (100%)	45 (90%)	0 (0%)	NA	<0.001
Lost to follow-up	8 (14%)	0 (0%)	4 (8%)	4 (100%)	NA	
Died	1 (2%)	0 (0%)	1 (2%)	0 (0%)	NA	
Intrathoracic	18 (31%)	2 (40%)	16 (32%)	0 (0%)	NA	<0.001
Extra-thoracic	7 (12%)	0 (0%)	7 (14%)	0 (0%)	NA	
Both	4 (7%)	3 (60%)	1 (2%)	0 (0%)	NA	
Not defined	30 (51%)	0 (0%)	26 (52%)	4 (100%)	NA	

IQR: interquartile range; BCG: Bacillus Calmette-Guérin; TST: tuberculin skin test; QFT: QuantiFERON-TB Gold. Malnutrition defined as weight-for-age *z*-score < −2 for children under 10 years old and body mass index -for-age *z*-score < −2 for children above 10 years. Stunting was defined as height-for-age < −2. ^a^ Controls have no results on stunting because height measurements were not taken.

**Table 2:** FujiLAM results by study group.

	FujiLAM		
	Positive *n* (%)	Negative *n* (%)	Total
Confirmed TB	3 (60%)	2 (40%)	5
Unconfirmed TB	3 (6%)	47 (94%)	50
Unlikely TB	1 (25%)	3 (75%)	4
Controls	1 (5%)	19 (95%)	20
All	8 (10.1%)	71 (89.9%)	79

## Data Availability

Not applicable.
